# Metal concentrations in transitional and coastal waters measured by passive (Diffusive Gradients in Thin-films) and spot sampling: MONITOOL Project Dataset

**DOI:** 10.1016/j.dib.2024.110145

**Published:** 2024-02-02

**Authors:** José Germán Rodríguez, Stephane Guesdon, Isabelle Amouroux, María Jesús Belzunce-Segarra, Philippe Bersuder, Thi Bolam, Pedro Brito, Miguel Caetano, Inês Carvalho, Margarida M. Correia dos Santos, Alessandro Desogus, Gary R. Fones, Jean-Louis Gonzalez, Joana Larreta, Luc Lebrun, Barbara Marras, Brendan McHugh, Florence Menet-Nédélec, Iratxe Menchaca, Vanessa Millán Gabet, Carlos E. Monteiro, Natalia Montero, Martin Nolan, Fiona Regan, Marta Rodrigo, Nuno Rosa, Marco Schintu, Anne Schmitt, Debora Todde, Lee Warford, Blánaid White, Hao Zhang

**Affiliations:** a AZTI, Herrera Kaia, Portualdea z/g, 20110 Pasaia, Spain; b Ifremer, LITTORAL, Environmental Resources Laboratory (Pertuis Charentais), Avenue de Mus de Loup, 17390 La Tremblade, France; c Ifremer, Chemical Contamination of Marine Ecosystems Unit, Rue de l'Ile d'Yeu, 44300 Nantes, France; d 06240 Beausoleil, Alpes-Maritimes, France; e CEFAS, Centre for Environment, Fisheries and Aquaculture Science, Lowestoft Laboratory, Pakefield Road, Suffolk, Lowestoft NR33 0HT, UK; f Instituto Português do Mar e da Atmosfera (IPMA), Division of Oceanography and Marine Environment, *Av*. Dr. Alfredo Magalhães Ramalho, 6, 1495-165, Algés, Portugal; g Centro de Química Estrutural, Institute of Molecular Sciences, Instituto Superior Técnico, *Av*. Rovisco Pais, 1049-001 Lisboa, Portugal; h UNICA, Dipartimento di Scienze Mediche e Sanità Pubblica, Università degli studi di Cagliari, 09124 Cagliari, Italy; i University of Portsmouth, School of the Environment Geography and Geosciences, Burnaby Road, Portsmouth PO1 3QL, UK; j Ifremer, LITTORAL, Environmental Resources Laboratory (Provence-Azur-Corse), Zone Portuaire de Brégaillon, 83507 La Seyne/mer, France; k Ifremer, LITTORAL, Environmental Resources Laboratory (Bretagne Occidentale), Place de la Croix - 29900 Concarneau, France; l Marine Institute, Rinville, Oranmore, Galway, Ireland; m Ifremer, LITTORAL, Environmental Resources Laboratory (Normandie), Avenue du Général de Gaulle, 14520 Port‑en‑Bessin, France; n ITC, Instituto Tecnológico de Canarias, Playa de Pozo Izquierdo, S/N. CP: 35119, Sta. Lucía, Las Palmas, Spain; o DCU Water Institute, Dublin City University, Dublin 9, Ireland; p Ifremer, LITTORAL, Environmental Resources Laboratory (Morbihan Pays de Loire), Rue de l'Ile d'Yeu, 44300 Nantes, France; q Lancaster Environment Centre, Lancaster University, Lancaster LA14YQ, UK

**Keywords:** Stripping voltammetry, Inductively coupled plasma mass spectrometry, Harbour, Metal speciation, EU Water Framework Directive, monitoring, seawater, DGT

## Abstract

The MONITOOL project (2017–2023) was carried out to describe the relationships between total dissolved and labile metal concentrations measured in spot water samples and in concurrently deployed Diffusive Gradients in Thin-films (DGTs) passive samplers, respectively. The ultimate aim was to adapt existing marine metal Environmental Quality Standards (EQS _marine water_) for DGTs, enabling their use in the context of the European Directives (the Water Framework Directive (WFD) and the Marine Strategy Framework Directive (MSFD)). Time-integrated metal concentrations provided by DGTs, representing several days, are an advantage compared to conventional spot sampling, especially in highly dynamic systems, such as transitional waters. Hence, the MONITOOL project aimed to provide a robust database of dissolved and labile metal concentrations in transitional and coastal waters, based upon co-deployments of DGTs and collection of spot water samples at several sampling sites (England, France, Ireland, Italy, Northern Ireland, Portugal, Scotland and Spain), followed subsequently by DGT and water metal analysis. Samplings were carried out in 2018 and 2022, following agreed protocols developed in the framework of the project.

The MONITOOL dataset includes metal concentrations from DGTs, measured with Inductively Coupled Plasma Mass Spectrometry (ICP-MS: Cd, Co, Cu, Fe, Mn, Ni, Pb, Zn) and in concurrently collected spot water samples by ICP-MS (Al, Cd, Co, Cu, Mn, Ni, Pb, Zn) and Anodic/Cathodic Stripping Voltammetry (ASV/CSV: Cd, Pb, Ni). Moreover, data on seawater physical-chemical parameters (salinity, temperature, dissolved oxygen, pH, turbidity, total suspended solids, dissolved organic carbon, and total organic carbon) is provided.

This database presents the results obtained using, concurrently, different forms of sampling and analytical techniques, enabling the comparison of the results obtained by these strategies and allowing the adaptation of EQS in marine water (EQS _marine water)_ to DGTs (EQS _DGT_), in the context of the WFD. Moreover, due to the large number of sampling sites, it could also be used for other types of research, such as those dealing with metal speciation or the determination of baseline levels.

Specifications Table

**Table:** 

Subject	Environmental chemistry		
Specific subject area	Assessment of the chemical status of transitional and coastal waters within the EU Water Framework Directive using passive sampling devices – adaptation of EQS_marine water_ to DGTs (EQS_DGT_)		
Data format	Raw		
Type of data	Table		
Data collection	DGT samplers were deployed for several days at each sampling site. Concurrently, discrete water sampling (spot sampling) was carried out. Metal (Cd, Co, Cu, Fe, Mn, Ni, Pb, Zn) concentrations in DGTs were measured by ICP-MS at IFREMER (France). The determination of conditional labile Pb and Cd concentrations (by ASV) and total dissolved Ni concentration (by CSV after UV irradiation) was carried out at IST (Portugal). Total dissolved concentrations (Al, Cd, Co, Cu, Mn, Ni, Pb, Zn) in filtered water samples were determined by an online pre-concentration seaFAST system coupled with an ICP-MS at IPMA (Portugal).		
Data source location	Station name	Longitude (°)	Latitude (°)
	LUZ	−15.426977	28.132998
	JINAMAR	−15.411215	28.042595
	GANDO	−15.373484	27.935261
	TALIARTE	−15.369056	27.990005
	TAGUS	−9.234432	38.695498
	SESIMBRA	−9.114133	38.435200
	PORTO	−8.704833	41.178405
	AVEIRO	−8.639185	40.725518
	M69	−8.340145	51.880607
	M70	−8.204457	51.881167
	ABW	−6.221505	53.348217
	POOLBEG MARINA	−6.216300	53.343500
	DUN LAOGHAIRE HARBOUR	−6.135100	53.296200
	DUBLINBAYBUOY4	−6.070000	53.293300
	DUBLINBAYBUOY2	−6.040950	53.327239
	BELFAST	−5.873650	54.635533
	38A	−5.629722	53.788889
	FAL	−5.027644	50.216220
	NEYLAND MARINA	−4.942176	51.712903
	TERENEZ	−4.268346	48.279809
	LIVERPOOL	−3.379333	53.533983
	DEBA	−2.356183	43.293664
	SAINT-NAZAIRE	−2.199419	47.280006
	HERRERA	−1.930883	43.322067
	MUSEO	−1.924500	43.329197
	PRACTICOS	−1.922817	43.325019
	LEZO2	−1.910900	43.322500
	LEZO	−1.910583	43.323008
	SAUMONARD	−1.255020	46.002055
	FONTENELLE	−1.109155	45.976029
	PORT-EN-BESSIN	−0.754130	49.347401
	MOLORINASCITA	9.104132	39.211338
	MOLO SABAUDO	9.107311	39.210304
	MOLODOGANA	9.113530	39.210849
	MOLOICHNUSA	9.114162	39.206636
	SANTELMO	9.126854	39.200633
	PARCO DI MOLENTARGIUS	9.211896	39.229907
Data accessibility	Repository name [1]: Rodriguez, J German; Guesdon, Stephane; Amouroux, Isabelle; Belzunce Segarra, Maria Jesus; Bersuder, Philippe; Bolam, Thi; Brito, Pedro; Caetano, Miguel; Carvalho, Inês; Correia dos Santos, Margarida M.; Desogus, Alessandro; Fones, Gary R.; Gonzalez, Jean-Louis; Larreta, Joana; Marras, Barbara; McHugh, Brendan; Menet-Nédélec, Florence; Menchaca, Iratxe; Millán Gabet, Vanessa; Monteiro, Carlos Eduardo; Montero, Natalia; Nolan, Martin; Regan, Fiona; Rodrigo Sanz, Marta; Rosa, Nuno; Schintu, Marco; Todde, Debora; Warford, Lee; White, Blánaid; Zhang, Hao (2023), “MONITOOL Project: dataset of metal concentrations in seawater from European estuaries and coastal sites measured by Diffusive Gradients in Thin-films (DGT) passive samplers and spot sampling.”, Mendeley Data, V1, doi:10.17632/r6fsncgyh7.1		
	Data identification number: *(or DOI or persistent identifier)* DOI:10.17632/r6fsncgyh7.1		
	Direct URL to data: https://data.mendeley.com/datasets/r6fsncgyh7/1		

## 1 Value of the Data

• This is the largest dataset in Europe providing DGT-labile metal concentrations and dissolved metal concentrations obtained concurrently and covering a large geographical scale.
• These data provide information on the seawater metal concentrations measured by different methods (passive sampling and spot sampling) concurrently, allowing intercomparison of the results obtained with the different methodologies in the framework of environmental monitoring studies.
• This data could be used for research dealing with European Directives and those promoting the acceptance of new sampling techniques and the development of EQS for passive sampling.
• This data can bring additional value to the research on metal speciation in seawater.
• This data can be used for baseline studies on the levels of dissolved metals in seawater.

## 2 Data Description

**Fig. 1:**
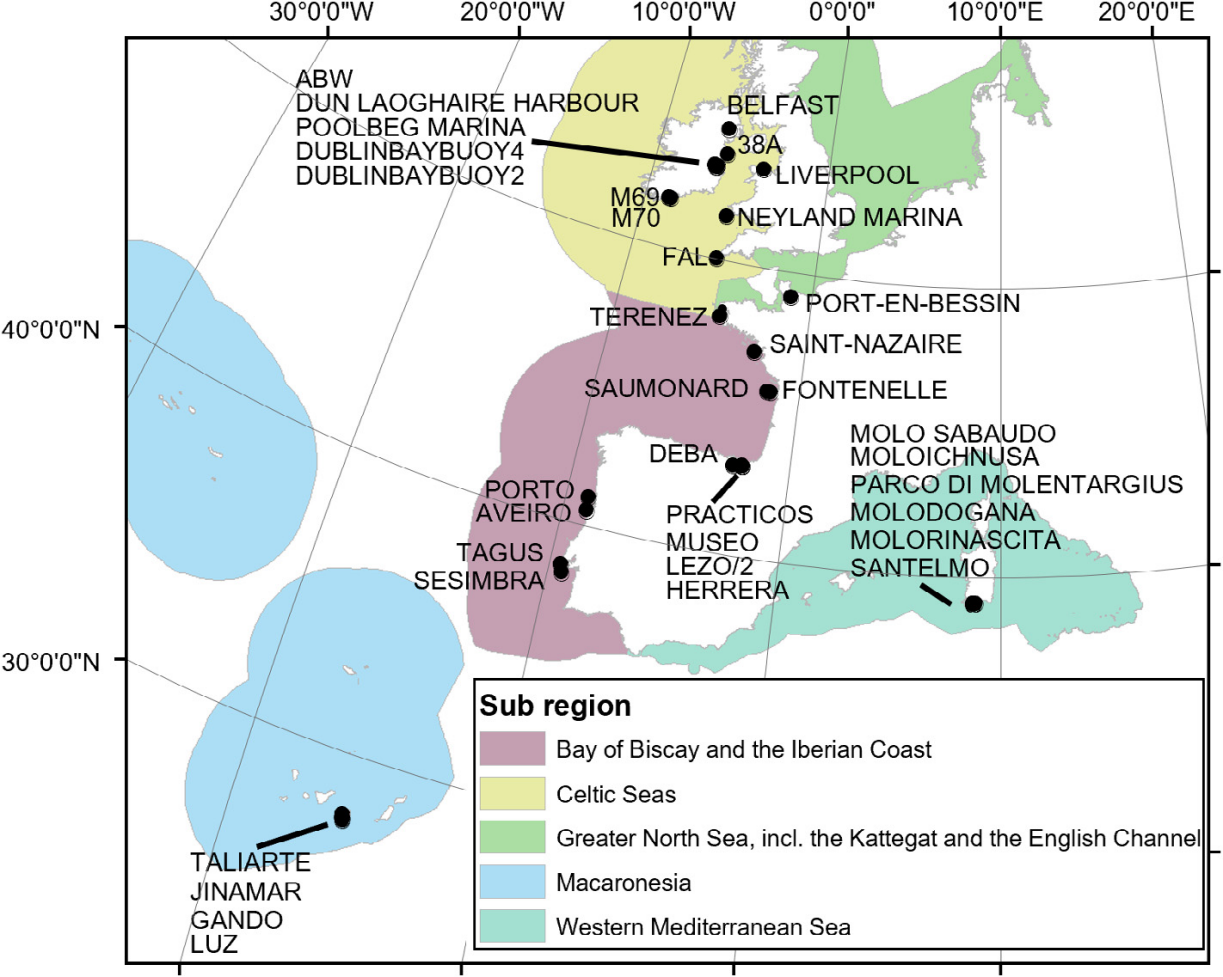
Location of sampling sites. Sub-regions according to the EU Marine Strategy Framework Directive (MSFD).

The dataset includes the information from field surveys carried out at 37 sampling sites (Fig. 1). Sampling sites correspond to the following ecoregions: Canary Islands, Iberian Coast, Bay of Biscay and English Channel, Celtic Sea, North Sea and Western Mediterranean Sea.

This database can be handled with any standard database manager or spreadsheet software. There are 24 fields (columns) whose information is detailed below.

**Label:** It provides a unique value that identifies the row (it has no additional meaning).

**Station:** Sampling station name. A description for most of the sites is available at [2].

**Longitude:** Longitude of the sampling station, in decimal degrees.

**Latitude:** Latitude of the sampling station, in decimal degrees.

**Sampling Campaign:** There were three sampling campaigns, which were named: ‘2018 Wet Season’, ‘2018 Dry Season’, and ‘2022 MONITOOL EXT’. It should be noted that not all sampling sites were sampled in the three campaigns.

**Sampling Date, Sampling Time** and **Sampling Time zone:** In the case of spot sampling, it refers to the day/time when the data or water sample was collected or recorded. In the case of time-integrated sampling with passive samplers (Diffusive Gradients in Thin Films (DGT)), it refers to the time when the passive sampler was retrieved.

**Sampling DGT Deployment time (days):** This field only has information linked to passive samplers. This refers to the number of days the passive sampler was deployed in water. The date and time of the start of deployment of the passive sampler is not given but can be calculated indirectly from available information, as indicated in the repository.

**Sampling Research centre:** This indicates the research institution that carried out the sampling.

**Sampling Level depth (m):** This indicates the depth of water sampling, *in situ* measurement or at which the passive sampler was deployed.

**Sampling tide (low, high, no tide):** In some cases, it is indicated whether the sampling was done relatively close to high tide (HT) or low tide (LT). It should be noted that some sampling sites were sampled twice on each date, while others were sampled only once.

**Sampling sample matrix:** In all cases it refers to ‘Raw water body’.

**Result method:** It presents 11 values that capture different types of information: i) ‘Diffusive Gradients in Thin Films (DGT)’: it refers to the metal content determined in passive samplers by inductively coupled plasma mass spectrometry; ii) ‘Spot sampling ICP-MS’: it refers to the metal content determined in spot samples by inductively coupled plasma mass spectrometry; iii) ‘Spot sampling voltammetry’: it refers to the metal content determined in spot samples by anodic/cathodic stripping voltammetry; iv) ‘Seawater temperature’: it refers to the seawater temperature measured *in situ*; v) ‘Seawater salinity’: it refers to the seawater salinity measured *in situ*; vi) ‘Seawater pH’: it refers to the seawater pH measured *in situ*; vii) ‘Seawater oxygen’: it refers to the concentration of dissolved oxygen (measured *in situ)*, viii) ‘Seawater turbidity’: it refers to the turbidity value (measured *in situ* or in the laboratory); ix) ‘Seawater dissolved organic carbon’: it refers to the dissolved organic carbon in seawater (measured in the laboratory); x) ‘Seawater total organic carbon’: it refers to the total organic carbon in seawater (measured in the laboratory); and xi) ‘Seawater suspended solid’: total suspended solids in seawater (measured in the laboratory).

**Result Details on the methodology:** a brief description is provided of which method or instrument was used to measure the value indicated in that row.

**Result Parameter:** it indicates for which metal concentration is provided or for which variable the result is given.

**Result Unit:** it indicates the units in which the result of the row is provided.

**Result Laboratory or research centre:** it indicates the Institution/laboratory that measured the value in that row.

**Result Precision (<, >, =):** it is complementary to the value in column ‘Result Value’. When ‘Result Precision’ is ‘=’, the result corresponds to the value displayed in ‘Result value’. When ‘Result Precision’ is ‘<’, the result is less than the value displayed in ‘Result value’; in this case, the value corresponds to the quantification limit of the device or method. When ‘Result Precision’ is ‘>’, the result is greater than the value displayed in ‘Result value’; in this case, the result is outside the validated range of values of the device or the method.

**Result value:** it is the measured value and should be combined with ‘Result Unit’ and ‘Result Precision’.

**Result Uncertainty:** this refers to the measurement uncertainty in the metal content determined in spot samples measured by voltammetry.

**Relative combined standard uncertainty (Nordtest approach,%):** this refers to the measurement uncertainty in the metal content determined in spot samples measured by ICP-MS.

**Result Quality level or remarks:** This field is used to note indications that may be relevant for the interpretation of the results displayed in the row.

**Result Field Replicate:** This field is only used in those rows with metal results obtained by passive samplers (DGT) or measured voltammetry in spot samples.

## 3 Experimental Design, Materials and Methods

### 3.1 Passive sampling

The passive samplers used were the LSNM-NP Loaded DGT devices for cationic trace metals in waters consisting of a standard DGT plastic holder with a polyethersulphone filter membrane (0.45 μm pore size), 0.8 mm agarose cross-linked polyacrylamide (APA) diffusive gel and Chelex® binding layer (DGT® Research Ltd, Lancaster, UK; reference: LSNM-NP open-pore Loaded DGT device for metals (A) in solution). The exposure time, *i.e.*, the deployment time of the passive sampler in water, varied from 2 to 15 days. In most cases, DGTs were used in triplicate. Details on DGT handling guidelines, including DGT pre-deployment storage, DGT assembly, DGT deployment/retrieval and transport and DGT dismantling and pre-analysis extractions, are given in [3]. For further guidance and images of the sampling campaigns performed in the framework of the MONITOOL project, it is recommended to read [4].

Details of the analytical method used for measuring the accumulated metal content (Cd, Co, Cu, Fe, Mn, Ni, Pb, Zn) in DGTs and for the back-calculation of metal concentrations in seawater are given in [2]. It should be noted that all DGT analyses were performed in the same laboratory (IFREMER, France).

### 3.2 Spot sampling for voltammetry and ICP-MS analyses

Concurrently with the exposure of DGTs, spot water samples were collected at the same depth with Niskin bottles or directly using collection flasks. The frequency of spot sampling varied between sites. The water samples for voltammetric analysis were filtered (DigiFILTER with 0.45 µm pore size Teflon membrane) and acidified on site (or as soon as practicable) and kept refrigerated until analysis. The samples for ICP-MS analysis collected in 2018 were frozen and sent to a single laboratory (CEFAS, UK), where they were filtered (DigiFILTER with 0.45 µm pore size Teflon membrane) and acidified. The samples for ICP-MS analysis collected in 2022 were filtered (DigiFILTER with 0.45 µm pore size Teflon membrane) and acidified on site (or as soon as practicable) and kept refrigerated until analysis.

Detailed information about the determination of conditional labile Pb and Cd concentrations, by ASV, and total dissolved Ni concentration, by CSV after UV irradiation, and the total dissolved concentrations (Al, Cd, Co, Cu, Mn, Ni, Pb, Zn) by an online pre-concentration seaFAST system coupled with an ICP-MS can be found in [2]. It should be noted that all metal analyses in spot water samples were performed in the same laboratories: IST (Portugal) in the case of the voltammetric analysis, and IPMA (Portugal) in the case of ICP-MS.

### 3.3 Spot sampling for other laboratory analyses

In some cases, complementary water samples were taken for the analysis of dissolved organic carbon, turbidity, etc. It should be noted that, unlike the analytical determinations of metals, the same procedure was not always used to determine these complementary variables. A description of the methods is given in the database.

### 3.4 *In situ* measurement of other variables

After the collection of each water sample, salinity, temperature, oxygen concentration, etc., were measured *in situ*, usually with multiparameter probes, as described in the database. It should be noted that the probes varied between the laboratories performing the sampling campaigns.

## Limitations

Not applicable.

## Ethics Statement

The authors have read and follow the ethical requirements for publication in Data in Brief and confirm that the current work does not involve human subjects, animal experiments, or any data collected from social media platforms.

## CRediT Author Statement

**José Germán Rodríguez:** Writing, Original draft preparation, Investigation, Data curation. **Stephane Guesdon:** Writing - Review & Editing, Data curation, Investigation, Methodology (Database model conceptualization). **Isabelle Amouroux:** Investigation, Data Curation, Writing - Review & Editing, Project administration, Funding acquisition. **María Jesús Belzunce-Segarra:** Investigation, Writing-Review & Editing, Project administration, Funding acquisition. **Philippe Bersuder:** Writing - Review & Editing, Data curation, Investigation. **Thi Bolam:** Writing - Review & Editing, Data curation, Investigation, Funding acquisition. **Pedro Brito:** Investigation. **Miguel Caetano:** Investigation, Writing-Review & Editing, Project administration, Funding acquisition. **Inês Carvalho:** Investigation. **Margarida M. Correia dos Santos:** Investigation, Writing - Review & Editing, Project administration. **Alessandro Desogus:** Investigation. **Gary R. Fones:** Conceptualization, Writing-Review & Editing. **Jean-Louis Gonzalez:** Investigation, Writing-Review & Editing, Data curation, Project administration, Funding acquisition. **Joana Larreta:** Investigation, Writing-Review & Editing. **Luc Lebrun:** Investigation. **Barbara Marras:** Investigation, Writing-Review & Editing. **Brendan McHugh: :** Conceptualization, Writing-Review & Editing. **Florence Menet-Nédélec**: Investigation, Review & Editing. **Iratxe Menchaca:** Investigation, Writing-Review & Editing. **Vanessa Millán Gabet:** Investigation, Project administration, Funding acquisition. **Carlos E. Monteiro:** Investigation, Writing-Review & Editing. **Natalia Montero**: Investigation, Writing-Review & Editing. **Martin Nolan:** investigation. **Fiona Regan:** Investigation, Writing-Review & Editing, Project administration, Funding acquisition. **Marta Rodrigo:** Investigation, Project administration, Funding acquisition. **Nuno Rosa:** Investigation. **Marco Schintu:** Investigation, Writing-Review & Editing, Project administration, Funding acquisition. **Anne Schmitt:** Investigation. **Debora Todde**: Investigation. **Lee Warford:** Investigation, Writing-Review & Editing. **Blánaid White:** Investigation, Writing-Review & Editing, Project administration, Funding acquisition. **Hao Zhang:** Conceptualization.

## Data Availability

MONITOOL Project: dataset of metal concentrations in seawater from European estuaries and coastal sites measured by Diffusive Gradients in Thin-films (DGT) passive samplers and spot sampling (Original data) (Mendeley Data).
